# Antibacterial Activity and Molecular Docking Studies of Black Cumin (*Nigella sativa* L.) Oil and Its Comparison with Some Antibiotics

**DOI:** 10.3390/ijms27115074

**Published:** 2026-06-04

**Authors:** Ayşe Kanıcı Tarhane, Celal Tuğrul Zeyrek, Serdal Tarhane, Murat Sert, İbrahim Filazi, Fatih Büyük, Lütfiye Sirka, Çağlar Salduz

**Affiliations:** 1 Department of Pharmacology and Toxicology, Faculty of Veterinary Medicine, Kafkas University, Kars 36000, Türkiye; caglar.salduz@kafkas.edu.tr; 2 Institute of Nuclear Sciences, Ankara University, Beşevler 10. Yıl Campus, Beşevler, Ankara 06100, Türkiye; celaltugrulzeyrek@ankara.edu.tr; 3 Laborant and Veterinary Health Program, Veterinary Department, Şabanözü Vocational School, Çankırı Karatekin University, Çankırı 18100, Türkiye; serdaltarhane@karatekin.edu.tr; 4 Vocational School of Health Services, Ankara Yıldırım Beyazıt University, Ankara 06300, Türkiye; msert06@gmail.com; 5 Central Research Laboratory Application and Research Center (ÇANKAM), Çankırı Karatekin University, Çankırı 18100, Türkiye; ibrahimfilazi@karatekin.edu.tr; 6 Department of Microbiology, Faculty of Veterinary Medicine, Kafkas University, Kars 36300, Türkiye; fatihbyk08@hotmail.com; 7 Department of Medical Services and Techniques, Şabanözü Vocational School, Çankırı Karatekin University, Çankırı 18100, Türkiye; lutfiyesirka@karatekin.edu.tr

**Keywords:** antibacterial activity, beta-lactam antibiotics, black cumin (*Nigella sativa* L.) oil, molecular docking, thymoquinone

## Abstract

Black cumin (*Nigella sativa* L.) oil has been traditionally used to manage infectious diseases. The scientific validation of its antibacterial potential remains of significant pharmacological interest. This study evaluated the in vitro antibacterial activity of cold-pressed black cumin oil against selected bacterial strains and compared its efficacy with that of common β-lactam antibiotics, supplemented by mechanistic insight through molecular docking. Pure oil was obtained via cold-press extraction from seeds. Antibacterial activity was evaluated using the disk diffusion and broth microdilution methods against *Staphylococcus aureus* NCTC10788, *Bacillus cereus* NCTC7464, *Listeria monocytogenes* ATCC11994, *Escherichia coli* NCTC2001, and *Salmonella typhimurium* NCTC11994. Commercial antibiotic disks containing cloxacillin (5 µg), cefoperazone (75 µg), penicillin (40 µg), and amoxicillin (25 µg) served as a reference. Potential molecular interactions were explored by the density functional theory (DFT) optimization of thymoquinone at the B3LYP/6-31+G(d,p) level, followed by molecular docking against bacterial targets. Inhibition zone diameters ranged from 13.5 ± 0.7 mm to 34 ± 2.1 mm, and minimum inhibitory concentration (MIC) values varied between 6.7 ± 2.3 and 64 ± 0.0 µg/mL depending on the bacterial strain tested. Black cumin oil demonstrated a stronger inhibitory effect on *B. cereus* and *L. monocytogenes* than the other bacteria tested, and exhibited a significantly higher inhibitory effect than some of the antibiotics tested (*p* < 0.05). In contrast, no statistically significant differences were observed among treatments against *E. coli* (*p* > 0.05). Overall, Gram-positive bacteria showed greater susceptibility to black cumin oil than Gram-negative bacteria. The computational analyses demonstrated stable binding interactions supporting the experimental results. These integrative in vitro and in silico findings provide mechanistic evidence for the traditional use of black cumin oil in treating infections. The results suggest that black cumin oil could be a promising natural antibacterial candidate; however, further toxicological and pharmacokinetic evaluations are required prior to clinical use.

## 1. Introduction

Bacterial resistance to antibiotics is one of the major public health challenges of the modern era [1]. The uncontrolled and excessive use of antibiotics has accelerated the development of resistance among pathogenic microorganisms. The increasing resistance exhibited by bacteria is gradually pushing humanity back toward the pre-antibiotic era. For this reason, the investigation of alternative and natural antibacterial agents has gained critical importance. Historical records indicate that plants have been used for the treatment of various diseases for thousands of years [2,3]. The development and production of pharmaceuticals from medicinal plants began with the direct isolation of primary compounds from plant materials. Among these plants, black cumin (*Nigella sativa* L.) is one of the most extensively studied due to its wide spectrum of naturally occurring bioactive constituents [4].

Black cumin oil contains several biologically active components, including thymoquinone, thymohydroquinone, phenolic compounds, and volatile oils, which have been reported to possess antioxidant, anti-inflammatory, antimicrobial, antidiabetic, antihypertensive, antiasthmatic, antitumor, neuroprotective, gastroprotective, diuretic, food-preserving, and lactation-enhancing properties [2,5,6,7,8,9,10,11,12,13,14].

The compounds found in black cumin oil are known to alter bacterial cell permeability, interfere with enzymes involved in energy production, disrupt protein layers in the cell wall, and ultimately induce bacterial cell death [15]. Yaman et al. [16] reported that black cumin accelerates wound healing, mainly due to its antioxidant effects, and that thymoquinone prevents lipid peroxidation within cell membranes. Similarly, Hadi et al. [17] suggested that black cumin oil may alleviate inflammation in rheumatoid arthritis, reduce oxidative stress biomarkers such as malondialdehyde (MDA) and nitric oxide (NO), improve swelling and tenderness, and serve as a supportive therapeutic option for affected patients. Pengzhan et al. [18] demonstrated that thymoquinone, the major active component of black cumin, inhibits gastric cancer cell proliferation and triggers apoptosis and autophagy in a concentration-dependent manner.

Animal studies have shown that black cumin oil is generally safe, although toxicity may occur at very high doses. With its low toxicity and broad pharmacological activity, black cumin is considered a promising natural product for modern medicine [19].

Despite extensive pharmacological investigations having been conducted on *Nigella sativa*, only a limited number of studies have comprehensively integrated in vitro antibacterial assays with in silico molecular docking analyses and comparative evaluations against commonly used β-lactam antibiotics to elucidate the possible antibacterial mechanisms of its major bioactive constituents against Gram-positive and Gram-negative foodborne pathogens. Therefore, combining experimental antibacterial assays with molecular docking approaches may provide a more comprehensive understanding of the inhibitory potential of black cumin oil and the molecular interactions underlying its antibacterial activity. Furthermore, molecular docking studies are a valuable tool in drug discovery, as they enable the investigation of potential therapeutic compounds within a short time frame at very low costs.

In this study, the in vitro antibacterial activity of black cumin oil against various bacterial species was evaluated by performing the disk diffusion method and minimum inhibitory concentration (MIC) tests, and its efficacy was compared with that of selected antibiotics. These findings were further supported and interpreted through molecular docking analyses. In this context, the protein targets selected for molecular docking—DNA gyrase (*S. aureus*, PDB ID: 3HO8), β-lactamase (*B. cereus*, PDB ID: 5V8E), dihydrofolate reductase (*E. coli*, PDB ID: 1T7D), listeriolysin O (*L. monocytogenes*, PDB ID: 1AOD), and SopB effector protein (*S. typhimurium*, PDB ID: 8T0J)—were chosen because they represent key bacterial enzymes and virulence factors involved in DNA replication, antibiotic resistance, folate metabolism, host cell invasion, and pathogenicity. Thereby, a mechanistic basis was provided to evaluate the antibacterial potential of thymoquinone.

## 2. Results

### 2.1. Gas Chromatography–Mass Spectrometry (GC-MS) Analysis of Black Cumin Oil Composition

**Figure 1:**
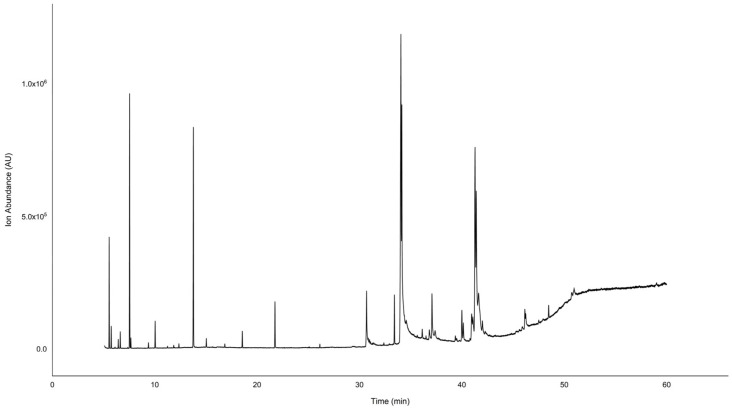
GC-MS total ion chromatogram (TIC) of black cumin oil.

The compounds detected in black cumin oil by GC-MS were manually checked and tentatively identified by comparing the acquired mass spectra with the National Institute of Standards and Technology (NIST 05) database. Relative abundances were calculated from chromatographic peak areas and expressed as area percentages (%) (Figure 1; Table 1). Linoleic acid, oleic acid, 1-glyceryl linoleate, β-monoolein, thymoquinone, and p-cymene were the main constituents detected. The predominance of linoleic acid, oleic acid, and related lipid constituents is consistent with previous reports on cold-pressed black cumin seed oil, in which linoleic acid was described as the major fatty acid, followed by oleic acid and palmitic acid. The detection of thymoquinone is also consistent with previous reports identifying it as one of the characteristic bioactive constituents of black cumin oil [20,21,22]. These results provide a compositional profile of the tested oil based on relative GC-MS peak areas.

### 2.2. Calculation Details

The root-mean-square deviation (RMSD) values and binding energies from the docking studies of thymoquinone with *S. aureus*, *B. cereus*, *E. coli*, *L. monocytogenes*, and *S. typhimurium* are presented in Table 2. The interacting figures were generated using Discovery Studio Visualizer, Version 16.1.0.15350 [23].

### 2.3. Molecular Docking Studies of Thymoquinone

**Figure 2:**
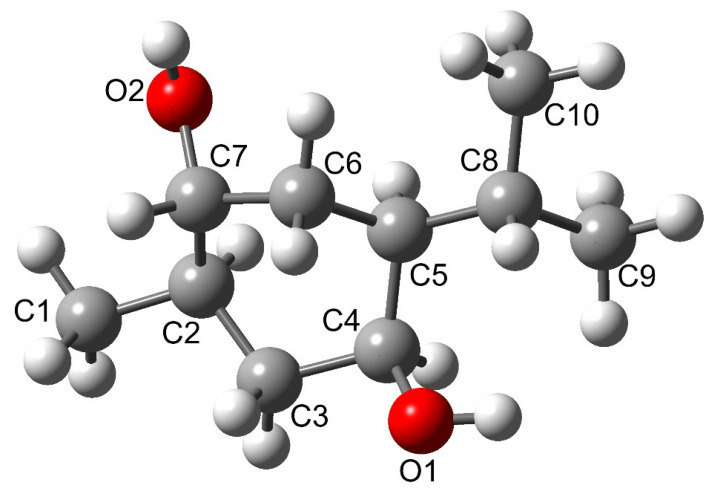
Optimized structure of thymoquinone at the B3LYP/6-31+G(d,p) level of the DFT method.

The optimized geometrical parameters and structure of thymoquinone were given in Figure 2 and Table 3 at the DFT/B3LYP/6-31+G(d,p) level. The calculated values of the bond distances C4—O1 and C7—O3 are 1.4380 Å and 1.43905 Å, which indicate the single C–O bond character.

**Figure 3:**
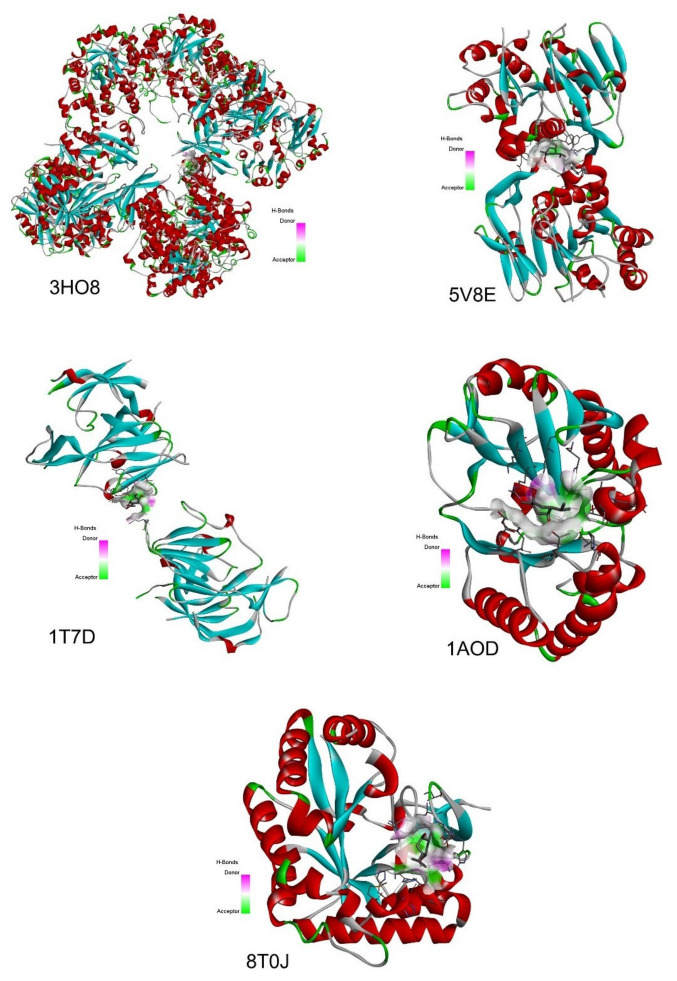
The energetically most favorable docked poses obtained from the rigid molecular docking of thymoquinone with 3HO8, 5V8E, 1T7D, 1AOD, and 8T0J.

**Figure 4:**
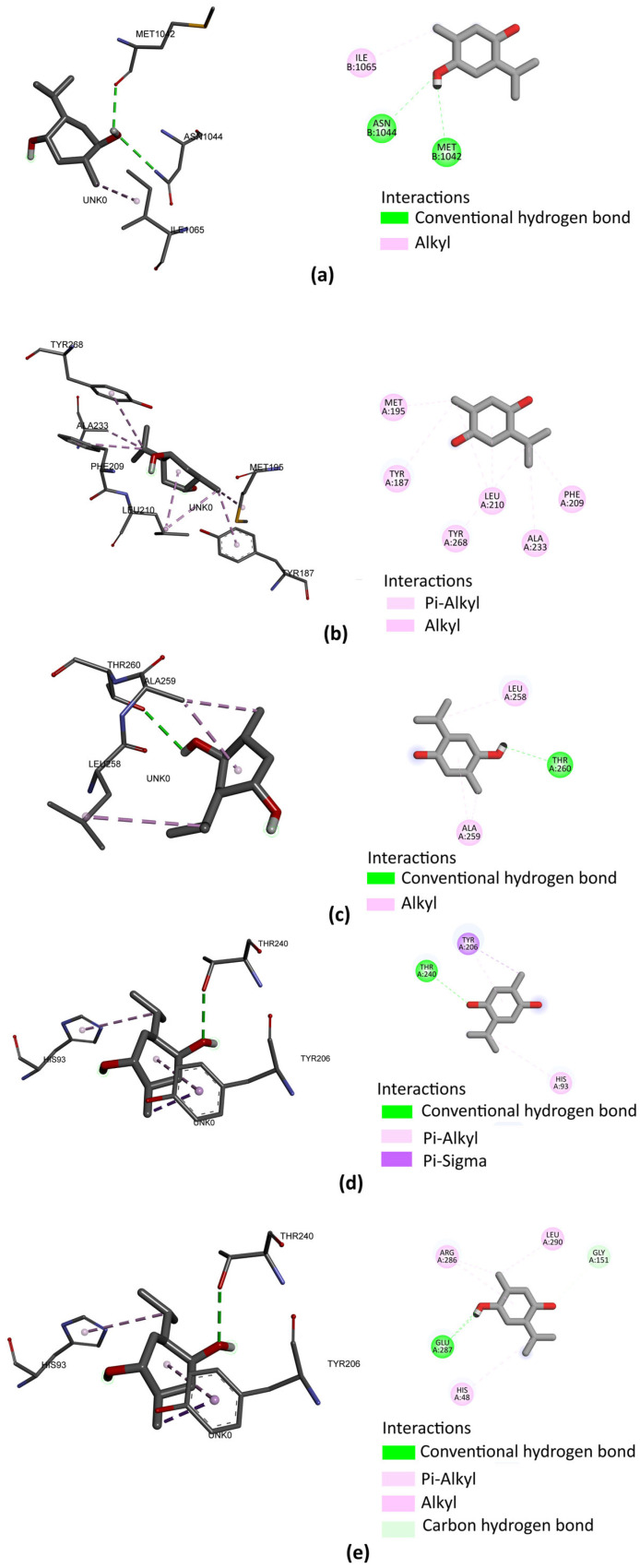
The non-covalent interactions of thymoquinone with (**a**) 3HO8, (**b**) 5V8E, (**c**) 1T7D, (**d**) 1AOD, and (**e**) 8T0J in 3D and 2D.

The molecular docking calculations of thymoquinone, aimed at explaining the binding interaction with the best inhibitory effect against *S. aureus* (PDB ID: 3HO8), *B. cereus* (PDB ID: 5V8E), *E. coli* (PDB ID: 1T7D), *L. monocytogenes* (PDB ID: 1AOD), and *S. typhimurium* (PDB ID: 8T0J), were performed by using the AutoDock-Vina software version 1.2.7 [24]. The RMSD values and bonding energies for each molecular docking study of thymoquinone are given in Table 2. The most convenient docked poses were obtained from the rigid molecular docking of thymoquinone with each of 3HO8, 5V8E, 1T7D, 1AOD, and 8T0J and are illustrated in Figure 3. The non-covalent interactions between thymoquinone with each of 3HO8, 5V8E, 1T7D, 1AOD, and 8T0J are also depicted in 3D and 2D in Figure 4. In the docking figures (Figure 4), the hydrogen bonds are indicated in green, alkyl contacts in pink, pi-alkyl contacts in light pink, pi-sigma contacts in purple and carbon–hydrogen bond interactions in mint colour. Interacting residues are labeled directly on the structures, and bond distances are provided in the legends for reference. This refinement reduces visual density and allows readers to more easily identify the critical binding features of thymoquinone across different bacterial protein targets. The interaction parameters such as binding sites, binding types and bond distances are listed in Table 4. The relative binding energy values were calculated as −4.6, −5.3, −4.9, −4.4 and −4.8 kcal/mol for the docking interactions of thymoquinone with 3HO8, 5V8E, 1T7D, 1AOD, and 8T0J, respectively. In our docking analysis, thymoquinone exhibited binding energies between −4.4 and −5.3 kcal/mol across the selected protein targets. Although these values reflect moderate affinities, they are within the range commonly reported for natural compounds in initial docking studies [25,26,27,28].

Thymoquinone was found to bind at the active sites of several bacterial proteins: MET1042, ASN1044, and ILE1065 of *S. aureus* (PDB ID: 3HO8); ALA233, LEU210, MET195, PHE209, TYR268, and TYR187 of *B. cereus* (PDB ID: 5V8E); THR260, LEU258, and ALA259 of *E. coli* (PDB ID: 1T7D); THR240, HIS93, and TYR206 of *L. monocytogenes* (PDB ID: 1AOD); and GLU287, HIS48, ARG286, LEU290, and GLY151 of *S. typhimurium* (PDB ID: 8T0J). The interactions included conventional hydrogen bonds (bond lengths ranging from 2.35 to 3.05 Å), alkyl contacts, pi-alkyl, pi-sigma, and carbon–hydrogen bonds, with each protein exhibiting a distinct combination of these interaction types. These results indicate that thymoquinone engages multiple binding motifs across different bacterial targets, consistent with its moderate affinity and potential contribution to antibacterial activity.

### 2.4. Study Limitations and Future Perspectives

While our study provides significant insights into the molecular interactions of thymoquinone with the target protein, we recognize that our approach has certain limitations. The in vitro experiments were conducted using total black cumin oil, which is a complex matrix consisting of various bioactive compounds, including p-cymene, carvacrol, and diverse fatty acids, as confirmed by our GC-MS analysis (Table 1) [29]. Molecular docking simulations in this study were focused primarily on thymoquinone, the major active constituent. Consequently, the biological activity observed cannot be solely attributed to thymoquinone, as the synergistic, additive, or potentially antagonistic interactions between thymoquinone and other minor constituents may play a crucial role in the overall therapeutic efficacy of the oil [30,31]. Future studies should employ a multi-ligand docking approach or molecular dynamics (MD) simulations of the whole-oil profile to elucidate these complex synergistic mechanisms, which would offer a more robust correlation between the chemical composition and the observed pharmacological activity. Taken together, these docking results suggest that thymoquinone contributes to the antibacterial activity of black cumin oil through moderate affinity interactions with key enzymatic residues, though the full therapeutic effect likely arises from synergistic interactions among multiple oil constituents.

### 2.5. Antibacterial Activity

The inhibition zone diameters and minimum inhibitory concentration (MIC) values obtained in this study are presented in Table 5.

The antibacterial activities of black cumin extract and the tested antibiotics against the bacterial isolates were evaluated by measuring the inhibition zone diameters. Two-way ANOVA revealed significant effects of both the bacterial species and antimicrobial agents on the inhibition zone diameters (*p* < 0.001). In addition, a significant interaction was observed between the bacterial species and antimicrobial treatments (*p* < 0.001), indicating that the effectiveness of the antimicrobial agents varied depending on the bacterial species tested. Among the tested agents, black cumin exhibited notable antibacterial activity, particularly against *B. cereus* and *L. monocytogenes*, with inhibition zones of 34.0 ± 2.1 mm and 22.0 ± 0.0 mm, respectively. In the case of *B. cereus*, black cumin showed significantly higher antibacterial activity than cloxacillin and penicillin (*p* < 0.05), while *B. cereus* was resistant to cefoperazone and amoxicillin. Similarly, black cumin demonstrated an inhibitory activity comparable to or greater than that of several antibiotics against *S. aureus*, *E. coli*, and *L. monocytogenes*. Tukey’s post hoc analysis indicated significant differences among the treatment groups within several bacterial species, as denoted by different superscripts (*p* < 0.05). However, no significant differences were observed among the treatments against *E. coli* (*p* > 0.05), where all antimicrobial agents produced similar inhibition zones. Overall, the results suggest that black cumin possesses a broad-spectrum antibacterial potential and, in some cases, may exhibit inhibitory effects comparable to those of conventional antibiotics (Table 5).

For *S. aureus*, the lowest MIC value was observed in the cefoperazone group (26.7 ± 9.2 µg/mL), which was significantly lower than the MIC values of the penicillin, cloxacillin, and amoxicillin groups (*p* < 0.05), indicating a higher antibacterial activity of cefoperazone against this strain. For B. cereus, black cumin oil and penicillin exhibited the lowest MIC values (6.7 ± 2.3 µg/mL and 13.3 ± 4.6 µg/mL, respectively), demonstrating a significantly stronger antibacterial activity compared to cefoperazone and amoxicillin (*p* < 0.05). No statistically significant difference was detected among the tested agents against E. coli (*p* > 0.05), where all treatments showed comparable MIC values. For *L. monocytogenes*, black cumin oil (6.7 ± 2.3 µg/mL) and penicillin (8 ± 0.0 µg/mL) showed the lowest MIC values, with black cumin exhibiting a significantly greater antibacterial activity than amoxicillin (*p* < 0.05). Against *S. typhimurium*, penicillin (6.7 ± 2.3 µg/mL) showed the lowest MIC value, followed by black cumin oil and cloxacillin (8 ± 0.0 µg/mL for both), all of which demonstrated a significantly higher activity than cefoperazone and amoxicillin (*p* < 0.05). Overall, the MIC values varied depending on the bacterial species, and statistical analysis confirmed significant differences among the treatment groups for *S. aureus*, *B. cereus*, *L. monocytogenes*, and *S. typhimurium* (*p* < 0.05). Notably, black cumin oil exhibited a strong antibacterial activity, particularly against Gram-positive bacteria such as *B. cereus* and *L. monocytogenes*. In contrast, no significant differences were observed among the treatments against E. coli, indicating uniform and limited variability in susceptibility (Table 6).

## 3. Discussion

Natural oils can inhibit or suppress the growth of bacteria, yeasts, and molds due to their various bioactive constituents. These compounds act on the bacterial cell wall and cytoplasm and may even cause significant alterations in cell morphology. Such mechanisms help explain the antibacterial activity of natural oils against pathogenic microorganisms. Numerous studies have confirmed the antimicrobial and therapeutic potential of black cumin oil [6]. However, the uncontrolled consumption of antimicrobial plant oils as dietary supplements may also exert antagonistic effects on probiotic microorganisms.

The present study demonstrated that black cumin oil possesses a broad-spectrum antibacterial activity against both Gram-positive and Gram-negative bacteria. The inhibition zone diameters ranged from 13.5 ± 0.7 mm to 34 ± 2.1 mm, while MIC values ranged from 6.7 ± 2.3 to 64 ± 0.0 µg/mL for the black cumin-treated groups. Among the tested strains, *B. cereus* exhibited the highest susceptibility, showing the largest inhibition zone diameter (34 ± 2.1 mm) together with one of the lowest MIC values (6.7 ± 2.3 µg/mL). Similarly, *L. monocytogenes* demonstrated high susceptibility to black cumin oil, whereas *E. coli* and *S. typhimurium* exhibited comparatively lower sensitivity in terms of inhibition zone diameters.

Nazzaro et al. [32] reported that bacterial morphology plays a role in determining the effectiveness of essential oils, noting that rod-shaped bacteria tend to be more susceptible than cocci. Consistent with this observation, black cumin oil produced strong antibacterial effects, particularly against *B. cereus* and *L. monocytogenes* in the present study. Similarly, Habib and Choudhry [33] demonstrated that thymoquinone extracted from black cumin seeds shows antibacterial activity against *Bacillus* species, supporting our findings.

Several previous studies have also reported the antibacterial efficacy of black cumin oil against both Gram-positive and Gram-negative bacteria [4,5,31,34,35,36]. Mehmood et al. [4] demonstrated that black cumin significantly inhibits the growth of various pathogenic bacteria, reporting findings that closely correspond with the results of the present study. In agreement with earlier reports, Gram-positive bacteria in our study generally exhibited greater susceptibility to black cumin oil than Gram-negative bacteria. This was evidenced by the larger inhibition zone diameters observed for *B. cereus* (34 mm), *L. monocytogenes* (22 mm), and *S. aureus* (17.5 mm) compared to *E. coli* (13.5 mm) and *S. typhimurium* (13.5 mm).

The higher susceptibility of Gram-positive bacteria to natural oils is generally attributed to differences in cell wall structure. The cell wall of Gram-negative bacteria is more complex, consisting of a thin peptidoglycan layer overlaid by an outer membrane that contains lipopolysaccharides (LPS) and various proteins. The LPS layer contributes to the resistance of Gram-negative bacteria against antimicrobial plant extracts. In contrast, the cell walls of Gram-positive bacteria facilitate the penetration of hydrophobic molecules, enabling these compounds to act on the cell wall and within the cytoplasm. Meanwhile, small hydrophilic molecules may pass through the outer membrane of Gram-negative bacteria, which partially explains why Gram-negative bacteria tend to be more resistant or less susceptible to hydrophobic antibiotics and toxic compounds.

One of the most important properties of essential oil components is their hydrophobic nature, which disrupts bacterial cell membranes and increases permeability [37]. Additionally, low pH conditions enhance the hydrophobicity of oils, facilitating their penetration through bacterial membranes and allowing them to reach intracellular targets [38]. However, the antimicrobial efficacy of oils also depends on their chemical composition; the position of functional groups within the molecules can significantly influence their activity [32,39]. Black cumin oil is rich in unsaturated fatty acids and bioactive compounds, conferring substantial pharmaceutical potential. It is important to note that the yield and bioactivity of these compounds are significantly affected by the extraction methods employed [40]. The pronounced antibacterial activity observed in this study, especially against *B. cereus* and *L. monocytogenes*, may therefore be associated with these bioactive constituents.

The comparative evaluation with conventional antibiotics further emphasized the antibacterial potential of black cumin oil. Against *B. cereus*, black cumin oil produced substantially larger inhibition zones than cloxacillin and penicillin, whereas cefoperazone and amoxicillin were ineffective under the tested conditions. In addition, black cumin oil exhibited one of the lowest MIC values against this bacterium, indicating strong inhibitory activity. For *L. monocytogenes*, black cumin oil generated the largest inhibition zone among all of the tested antimicrobial agents and demonstrated lower MIC values than cefoperazone and amoxicillin. Although cefoperazone exhibited the strongest activity against *S. aureus* based on the MIC values, black cumin oil still showed an antibacterial activity comparable to that of cloxacillin and amoxicillin. These findings indicate that black cumin oil may possess an antibacterial efficacy comparable to, and in some cases greater than that of certain conventional antibiotics.

Boka et al. [41] investigated the effects of black cumin supplementation on intestinal *E. coli* counts and epithelial cell morphology, reporting a reduction in *E. coli* populations with the dietary inclusion of black cumin. Similarly, Seidavi et al. [42] observed that adding 1–2% black cumin oil to the feed of laying hens, particularly during early production stages, reduced intestinal *E. coli* counts. Zouirech et al. [43] reported a promising antibacterial activity of black cumin oil against *E. coli* and all other tested strains. The inhibitory activity against *E. coli* observed in the present study supports these previous findings, although no statistically significant differences were detected among the tested antimicrobial agents.

Moreover, Zouirech et al. [43] noted that black cumin oil exhibits strong antioxidant, antibacterial, and antifungal activities, which are associated with its high thymoquinone content. They suggested that it could act synergistically with antibiotics and antifungals to reduce minimum inhibitory concentrations (MICs). Srinivasa et al. [44] reported that black cumin contains peptides and proteins with antimicrobial activity against bacteria and fungi in both in vitro and in vivo models, indicating potential contributions to novel drug development. The antibacterial effect of black cumin oil may also be attributed to the presence of thymol [45], which interacts with proteins, oxidized compounds, and enzymes, leading to phenolic toxicity [46].

Pachaiappan et al. [47] demonstrated that black cumin oil inhibited *S. aureus* growth at a concentration of 10 µg/mL, which is in agreement with the antibacterial activity observed in the present study. Similarly, Nair et al. [48] reported that black cumin oil exhibited a strong antibacterial activity against *L. monocytogenes*, producing a larger inhibition zone than gentamicin (*p* < 0.01). In the present study, black cumin oil produced a 22 mm inhibition zone against *L. monocytogenes*, exceeding those produced by all of the tested antibiotics and further confirming the strong antibacterial potential of the oil against this pathogen.

Although numerous studies have reported antibacterial effects of black cumin oil, contradictory findings have also been documented. Some studies demonstrated that black cumin oil failed to produce significant inhibitory effects against certain bacterial species or specific extract types [49,50,51].

Additionally, several studies have indicated that combinations involving black cumin may exert antagonistic or only limited synergistic effects with antibiotics, and that the observed outcomes can vary with bacterial species and osmotic conditions, thereby complicating generalized interpretations regarding its antibacterial efficacy [50,52,53].

In the present study, in addition to performing disk diffusion assays, MIC values were determined to provide a quantitative assessment of antibacterial activity, thereby strengthening the biological evaluation. While the integration of disk diffusion assays with DFT and docking analyses provides valuable preliminary insights into the antibacterial potential of thymoquinone, we acknowledge that the study remains largely descriptive. To strengthen the biological evaluation, future work will incorporate quantitative assays such as MIC and minimum bactericidal concentration (MBC) determinations, thereby providing more precise measures of antibacterial potency. On the computational side, MD simulations will be employed to validate the stability of thymoquinone–protein complexes over time, and binding free energy calculations (MM-PBSA/MM-GBSA) will be used to quantify interaction strength beyond static docking scores. Furthermore, multi-ligand docking approaches will be applied to the full chemical profile of black cumin oil to capture synergistic or antagonistic effects among its diverse constituents. These methodological enhancements will provide a deeper mechanistic understanding and a more robust correlation between chemical composition and observed pharmacological activity.

The DFT calculations provided an optimized geometry of thymoquinone for the use of the docking studies. The docking interactions observed between thymoquinone and these protein targets suggest that thymoquinone may interfere with bacterial DNA replication (via gyrase inhibition) [54], antibiotic resistance mechanisms (via β-lactamase binding) [55], folate metabolism (via DHFR inhibition) [56], and host invasion pathways (via SopB) [57]. This provides a mechanistic basis for the antibacterial activity of black cumin oil, although synergistic effects with other oil constituents must also be considered.

## 4. Materials and Methods

Black cumin seeds were purchased from a local commercial vendor in Çankırı province, Türkiye. Because the seeds were commercially sourced and not harvested from the wild, they were not stored. Black cumin oil was extracted from the seeds using a mechanical cold-pressing method without the use of heat or organic solvents. The resulting oil was filtered to remove residual particles and stored in sterile, dark-colored bottles at 4 °C until antimicrobial susceptibility testing. For antimicrobial assays, a stock solution of black cumin oil was prepared by dissolving the oil in Tween 20 (P2287, Sigma-Aldrich, Burlington, MA, USA) to obtain a concentration of 25.6 mg/mL.

### 4.1. GC-MS Analysis of Black Cumin Oil Composition

The chemical composition of black cumin seed oil was analyzed by GC-MS analysis using an Agilent 5975 mass spectrometer (Agilent Technologies, Santa Clara, CA, USA) coupled to an Agilent 7890 gas chromatograph (Agilent Technologies, Santa Clara, CA, USA). The chemical composition of the analytes was determined using data from the NIST library.

High-purity helium was used as the carrier gas at a constant flow rate of 1 mL/min. Separation procedures were performed on a J&W DB-5MS capillary column (50 m × 250 µm inner diameter × 0.25 µm film thickness). Samples were diluted with analytical-grade hexane at a ratio of 1:9 (*v*/*v*), and 1 µL of the prepared solution was injected using an automated liquid sampler. The injector temperature was set to 250 °C, and the injection was performed in split mode at a split ratio of 100:1.

The oven temperature program was initially held at 100 °C for 5 min, then increased to 250 °C at a rate of 5 °C/min and held at this temperature for 5 min. Subsequently, it was increased to 300 °C at the same rate and held for 10 min. The total run time was 60 min. The GC-MS transfer line temperature was set to 280 °C. The mass spectrometer was operated in the electron ionization mode at 70 eV, and data were acquired in the full-scan mode over an m/z range of 50–550. The solvent delay was 5 min. The ion source and quadrupole temperatures were 250 °C and 150 °C, respectively. The acquired mass spectra were manually inspected and compared with the NIST 05 mass spectral library for tentative compound identification. Relative abundances were calculated from chromatographic peak areas and expressed as area percentages.

### 4.2. Computational Methods and Molecular Docking Protocol

The molecular geometry of thymoquinone was first optimized using the density functional theory (DFT) method to obtain its most stable ground-state conformation for subsequent docking studies. Calculations were performed using the B3LYP/6-31+G(d,p) hybrid functional level within the Gaussian 09 software package, while GaussView version 6.1.1 was utilized for visualization [58,59]. Molecular docking simulations were then executed via AutoDock Vina [24] to investigate the binding affinities between thymoquinone and the target proteins. The 3D crystal structures of the target enzymes from *S. aureus* (PDB: 3HO8), *B. cereus* (PDB: 5V8E), *E. coli* (PDB: 1T7D), *L. monocytogenes* (PDB: 1AOD), and *S. typhimurium* (PDB: 8T0J) were retrieved from the Protein Data Bank (PDB). The protein structures selected for docking were chosen based on their biological relevance as antibacterial targets: DNA gyrase subunit B (3HO8, *S. aureus*), β-lactamase (5V8E, *B. cereus*), dihydrofolate reductase (1T7D, *E. coli*), listeriolysin O (1AOD, *L. monocytogenes*), and effector protein SopB (8T0J, *S. typhimurium*). These enzymes and virulence factors are essential for bacterial replication, resistance, or pathogenicity, making them suitable models for evaluating thymoquinone’s potential inhibitory effects. The preparation of the receptor files—including the addition of partial charges and the definition of rotatable bonds and torsion angles—was conducted using AutoDockTools (ADT) version 1.5.7 [60]. For each protein, a grid box of 30 × 30 × 30 Å was constructed, centered to encompass the key residues of the active site. The most favorable docking poses were determined based on the lowest binding free energy (ΔG) and RMSD values to ensure the reliability and quality of the docking results [60,61]. Although the docking simulations provided preliminary insights into the potential interactions of thymoquinone with bacterial protein targets, the binding energies obtained (−4.4 to −5.3 kcal/mol) indicate a moderate affinity typical of small natural ligands rather than strong inhibition. Docking alone cannot confirm inhibitory activity, and therefore these results should be interpreted cautiously. To validate the computational predictions, future studies will employ MD simulations to assess the stability of thymoquinone–protein complexes over time, and binding free energy calculations to provide quantitative estimates of interaction strength. In addition, enzyme inhibition assays will be conducted to experimentally confirm the computational findings and establish a direct correlation between docking predictions and biological activity.

### 4.3. Antibacterial Activity

The antibacterial activity of the oil was initially evaluated using the disk diffusion method, and the MIC values were subsequently determined by the broth microdilution method in accordance with the guidelines of the Clinical and Laboratory Standards Institute (CLSI, 2012 and 2024) [62,63]. All experiments were performed in triplicate, and the results were expressed as mean values ± standard deviation (SD).

The bacterial strains used in this study were *Staphylococcus aureus* NCTC10788, *Listeria monocytogenes* ATCC11994, *Bacillus cereus* NCTC7464, *Escherichia coli* NCTC2001, and *Salmonella typhimurium* NCTC11994. These strains were obtained from the Department of Microbiology, Faculty of Veterinary Medicine, Kafkas University. After preparing fresh cultures on appropriate solid media for 24–48 h, bacterial inocula were adjusted to a turbidity equivalent to the 0.5 McFarland standard (approximately 0.5 × 10^8^ CFU/mL) using nutrient broth (CM0001B, ThermoFisher Scientific, Waltham, MA, USA).

For the disk diffusion assay, 100 µL of each bacterial suspension was spread onto Mueller–Hinton (MH) Agar plates (ThermoFisher Scientific, CM0337B) using a sterile spreader. Blank sterile disks were impregnated with 20 µL of plant oil and placed onto the inoculated MH agar surfaces. Commercial antibiotic disks containing cloxacillin (5 µg), cefoperazone (75 µg), penicillin (40 µg), and amoxicillin (25 µg) were used as positive controls, whereas blank disks served as negative controls. The plates were incubated at 37 °C for 24 h, and the inhibition zone diameters were measured using a caliper.

The MIC values of the plant oil were determined using the broth microdilution method in sterile U-bottom 96-well microplates. The oil was dissolved in 0.5% Tween 20 to prepare a stock and serial two-fold dilutions were prepared in Mueller-Hinton Broth (MHB) (ThermoFisher Scientific, CM0405B), yielding final concentrations ranging from 256 to 0.25 µg/mL. Briefly, 100 µL of broth medium was added to each well, followed by 100 µL of the oil solution into the first well, and serial dilutions were performed across the plate.

To prepare the inoculum, bacterial suspensions adjusted to 0.5 McFarland standard were further diluted in MHB to obtain a final concentration of approximately 5 × 10^5^ CFU/mL in each well. Subsequently, 100 µL of the bacterial inoculum was added to all wells containing the diluted oil samples. Sterility control wells containing only broth medium and growth control wells containing broth plus bacterial suspension were included in each assay. Tween 20 was also tested as a negative control solvent.

The microplates were incubated at 37 °C for 18–24 h. Following incubation, bacterial growth was evaluated visually, and MIC values were defined as the lowest concentration of the oil that completely inhibited visible bacterial growth.

Commercial antibiotic powders of cloxacillin, cefoperazone, penicillin, and amoxicillin were also tested against the bacterial strains under the same broth microdilution conditions as the reference antimicrobial agents.

### 4.4. Statistical Analysis

All statistical analyses were performed using IBM SPSS Statistics version 32.0.0 and GraphPad Prism version 11.0.2. Inhibition zone diameter data were analyzed using two-way analysis of variance (Two-Way ANOVA) to evaluate the effects of the bacterial species and antimicrobial agents, as well as their interaction. Tukey’s multiple comparison test was applied for post hoc comparisons. For MIC data, one-way analysis of variance (One-Way ANOVA) was performed separately for each bacterial species to compare the antimicrobial agents. When significant differences were observed, Tukey’s post hoc test was used for pairwise comparisons. Data were expressed as mean ± standard deviation (SD), and differences were considered statistically significant at *p* < 0.05.

## 5. Conclusions

In conclusion, the increasing prevalence of antibiotic-resistant bacteria continues to represent a major global health concern, highlighting the urgent need for alternative and complementary antimicrobial agents. The in vitro findings of the present study demonstrated that black cumin oil possesses a broad-spectrum antibacterial activity against both Gram-positive and Gram-negative bacteria, with particularly strong inhibitory effects against *B. cereus*, *L. monocytogenes*, and *S. typhimurium*. In several cases, black cumin oil exhibited an antibacterial activity comparable to or greater than that of certain conventional antibiotics. Future studies focusing on antibiotic-resistant strains may further elucidate the potential of black cumin oil as a natural antimicrobial agent in modern therapeutic approaches. However, for clinical application, comprehensive in vivo studies along with toxicological and pharmacokinetic analyses are necessary. Special attention should be given to the oil’s chemical composition, synergistic interactions among its constituents, dosing, toxicity profile, and other pharmacological properties to fully understand its long-term efficacy and safety.

The geometry optimization and molecular docking of the compound thymoquinone with *S. aureus* (PDB ID: 3HO8), *B. cereus* (PDB ID: 5V8E), *E. coli* (PDB ID: 1T7D), *L. monocytogenes* (PDB ID: 1AOD), and *S. typhimurium* (PDB ID: 8T0J) were also investigated in this study. Conventional drug discovery methods are typically time-consuming and costly, whereas molecular docking allows for the rapid and cost-effective evaluation of potential therapeutics. Molecular docking analyses were performed in conjunction with experimental studies to elucidate the binding interactions of thymoquinone, which demonstrated the most potent inhibitory effects against *S. aureus*, *B. cereus*, *E. coli*, *L. monocytogenes*, and *S. typhimurium*.

This study demonstrates that black cumin, traditionally used in folk medicine, may also have a potential role in modern therapeutic applications.

## Figures and Tables

**Table 1:** GC-MS profile of black cumin oil, including the retention times and relative peak areas of the detected compounds.

Item No	RT (min)	Compound *	%Area **
1	5.550	β-thujene	3.04
2	5.751	α-pinene	0.68
3	6.437	Sabinene	0.28
4	6.632	(-)-β-pinene	0.51
5	7.547	p-cymene	8.07
6	7.673	D-limonene	0.35
7	10.042	cis-4-methoxythujene	0.95
8	13.773	Thymoquinone	8.26
9	15.043	Thymol	0.33
10	18.556	Longifolene	0.65
11	21.749	4-methoxy-2,3,6-trimethylphenol	1.77
12	30.693	Palmitic acid	3.80
13	33.411	6-epi-shyobunol	1.81
14	34.046	Linoleic acid	22.77
15	34.137	Oleic acid	14.60
16	36.123	Palmitic acid β-monoglyceride	0.46
17	37.078	Dipalmitin	3.66
18	40.002	β-Monolinolein isomer I	1.85
19	40.140	Oleic acid, 3-hydroxypropyl ester	1.03
20	40.958	β-Monolinolein isomer II	2.32
21	41.284	1-Glyceryl linoleate	13.34
22	41.399	β-Monoolein	8.57
23	46.142	Monolinoleoylglycerol derivative	0.91

RT: Retention time. * Compounds were tentatively identified by the comparison of acquired mass spectra with the NIST 05 library. ** Relative abundances are reported as chromatographic peak area percentages.

**Table 2:** Scoring results for the docking calculations of thymoquinone with 3HO8, 5V8E, 1T7D, 1AOD, and 8T0J by using AutoDock-Vina software.

Compound/PDB Code	Mode	Affinity (kcal/mol)	Distance from Best Mode	
			RMSD l.b.	RMSD l.b.
	1	−4.6	0.000	0.000
	2	−4.6	28.753	30.299
	3	−4.5	2.082	5.268
Thymoquinone -3HO8	4	−4.5	27.512	29.681
(*S. aureus*)	5	−4.4	28.387	30.021
	6	−4.3	3.094	5.252
	7	−4.3	25.552	26.900
	8	−4.3	4.155	6.027
	9	−4.3	1.400	4.651
	1	−5.3	0.000	0.000
	2	−5.1	5.299	6.662
	3	−5.1	14.808	16.188
Thymoquinone -5V8E	4	−5.1	3.182	4.578
(*B. cereus*)	5	−5.1	3.257	4.788
	6	−5.0	2.156	4.115
	7	−4.9	5.135	6.780
	8	−4.9	5.046	5.629
	9	−4.8	5.112	6.067
	1	−4.9	0.000	0.000
	2	−4.6	1.403	1.925
	3	−4.5	1.605	4.510
Thymoquinone -1T7D	4	−4.5	9.720	11.053
(*E. coli*)	5	−4.5	24.275	26.037
	6	−4.5	1.900	3.417
	7	−4.4	1.769	3.108
	8	−4.3	2.819	4.723
	9	−4.3	1.607	3.477
	1	−4.4	0.000	0.000
	2	−4.4	1.837	2.470
	3	−4.4	2.298	4.053
Thymoquinone -1AOD	4	−4.4	2.087	4.126
(*L. monocytogenes*)	5	−4.4	1.671	4.149
	6	−4.4	26.176	27.703
	7	−4.2	25.127	26.439
	8	−4.2	2.521	4.113
	9	−4.2	2.292	3.095
	1	−4.8	0.000	0.000
	2	−4.7	1.018	2.801
	3	−4.5	1.639	1.698
Thymoquinone -8T0J	4	−4.5	14.212	14.942
(*S. typhimurium*)	5	−4.5	26.451	27.524
	6	−4.4	26.911	27.693
	7	−4.3	26.653	27.525
	8	−4.3	2.361	3.748
	9	−4.2	10.674	12.381

PDB: Protein Data Bank., RMSD: Root-mean-square deviation.

**Table 3:** The bond lengths (Å), bond angles (°) and torsion angles (°) calculated for thymoquinone at the B3LYP/6-31+G(d,p) level.

Parameters	Calculated Values
Bond lengths (Å)	
C1—C2	1.53304
C1—C2	1.53478
C2—C3	1.54104
C4—O1	1.4380
C4—C5	1.54796
C5—C6	1.53776
C6—C7	1.54535
C7—O2	1.43905
C7—C2	1.54118
C5—C8	1.55040
C8—C9	1.54212
C8—C10	1.53979
Bond angles (°)	
C1—C2—C3	112.410
C2—C3—C4	113.280
C3—C4—O1	105.098
C3—C4—C7	105.098
C3—C4—C5	112.052
C4—C5—C6	108.013
C5—C6—C7	111.556
C7—C2—C3	109.642
C2—C7—O2	105.975
C6—C7—O2	118.810
C3—C4—O1	105.098
C5—C4—O2	112.904
Selected torsion angles (°)	
C1—C2—C7—O2	−80.826
C3—C2—C7—O2	153.728
C5—C6—C7—O2	−88.298
C2—C3—C4—O1	148.860
C6—C5—C4—O1	−82.461
C4—C5—C8—C9	55.801
C4—C8—C8—C10	178.305
C6—C5—C8—C9	−179.276
C6—C5—C8—C10	−56.772

**Table 4:** Binding interactions of the compound thymoquinone with *S. aureus* (PDB ID: 3HO8), *B. cereus* (PDB ID: 5V8E), *E. coli* (PDB ID: 1T7D), *L. monocytogenes* (PDB ID: 1AOD), and *S. typhimurium* (PDB ID: 8T0J).

Molecular Docking Study	Conventional Hydrogen Bonding Residues	Distance (Å)	Other Interaction Residues
(UNK0) [*]–3HO8	UNK0:H-B:MET1042:O B:ASN1044:ND2-UNK0:O	2.717 3.053	(UNK0:C-B:ILE1065) [a]
(UNK0) [*]–5V8E	-	-	(A:ALA233-UNK0:C) [a], (A:LEU210-UNK0) [a], (UNK0:C-A:LEU210) [a], (UNK0:C-A:MET195) [a], (A:PHE209-UNK0:C) [b], (A:TYR268-UNK0:C) [b], (A:TYR187-UNK0:C) [b]
(UNK0) [*]–1T7D	UNK0:H-A:THR260:OG1	2.774	(UNK0:C-A:LEU258) [a], (A:ALA259-UNK0) [a], (A:ALA259-UNK0:C) [a]
(UNK0) [*]–1AOD	A:THR240:OG1-UNK0:O	2.817	(A:HIS93-UNK0:C) [b], (A:TYR206-UNK0) [b], (UNK0:C-A:TYR206) [c]
(UNK0) [*]–8T0J	UNK0:H-A:GLU287:O A:GLU287:N-:UNK0:O	2.357 3.018	(A:HIS48-:UNK0:C) [b], (A:ARG286-UNK) [a], (UNK0:C-A:ARG286) [a], (UNK0:C-A:LEU290) [a], (A:GLY151:CA-UNK0:O) [d]

[*] Thymoquinone [a] Alkyl, [b] Pi-Alkyl, [c] Pi-Sigma [d] Carbon Hydrogen Bond.

**Table 5:** Antibacterial activity of black cumin oil and antibiotics against tested bacterial strains (mean ± SD).

Bacteria	Black Cumin	Cloxacillin	Penicillin	Cefoperazone	Amoxicillin
*S. aureus* (Gr+)	17.5 ± 0.7 ^a^	14.5 ± 0.7 ^b^	13.0 ± 1.4 ^b^	21.5 ± 0.7 ^a^	19.5 ± 0.7 ^a^
*B. cereus* (Gr+)	34.0 ± 2.1 ^a^	15.5 ± 0.7 ^b^	14.5 ± 0.7 ^b^	R	R
*E. coli* (Gr−)	13.5 ± 0.7 ^a^	12.0 ± 0.0 ^a^	13.5 ± 0.7 ^a^	14.5 ± 0.7 ^a^	15.5 ± 0.7 ^a^
*L. monocytogenes* (Gr+)	22.0 ± 0.0 ^a^	20.5 ± 0.7 ^a^	20.5 ± 0.7 ^a^	17.0 ± 1.4 ^b^	13.5 ± 0.7 ^c^
*S. typhimurium* (Gr−)	13.5 ± 0.7 ^b^	12.5 ± 0.7 ^b^	21.0 ± 0.0 ^a^	16.5 ± 0.7 ^b^	15.0 ± 0.0 ^b^

^a,b,c^ Different superscripts within the same row indicate statistically significant differences (*p* < 0.05). R: Resistant.

**Table 6:** Comparison of the antibacterial activities of black cumin and different antibiotics against various bacteria (µg/mL, mean ± SD) with ANOVA results.

Bacteria	Black Cumin	Cloxacillin	Penicillin	Cefoperazone	Amoxicillin	*p*
*S. aureus* (Gr+)	64.0 ± 0.0	53.3 ± 18.5	106.7 ± 37.0	26.7 ± 9.2	53.3 ± 18.5	<0.05
*B. cereus* (Gr+)	6.7 ± 2.3	32.0 ± 0.0	13.3 ± 4.6	256.0 ± 0.0	213.3 ± 73.9	<0.001
*E. coli* (Gr−)	64.0 ± 0.0	64.0 ± 0.0	64.0 ± 0.0	53.3 ± 18.5	64.0 ± 0.0	>0.05
*L. monocytogenes* (Gr+)	6.7 ± 2.3	13.3 ± 4.6	8.0 ± 0.0	16.0 ± 0.0	26.7 ± 9.2	<0.05
*S. typhimurium* (Gr−)	8.0 ± 0.0	8.0 ± 0.0	6.7 ± 2.3	16.0 ± 0.0	16.0 ± 0.0	<0.001

## Data Availability

The original contributions presented in this study are included in the article. Further inquiries can be directed to the corresponding authors.

## Abbreviations

The following abbreviations are used in this manuscript:

Å	Ångström (10^−10^ m), unit of length
A, B	Protein chain identifiers in PDB structures
ADT	AutoDockTools
ALA	Alanine
ARG	Arginine
ASN	Asparagine
ATCC	American Type Culture Collection
AutoDock-Vina	Molecular docking software
B3LYP	Becke three-parameter Lee–Yang–Parr functional
CFU	Colony Forming Unit
CLSI	Clinical and Laboratory Standards Institute
DFT	Density Functional Theory
EMV	Electron Multiplier Voltage (mode/setting)
GC-MS	Gas Chromatography–Mass Spectrometry
GLU	Glutamic acid
GLY	Glycine
H	Hydrogen atom
HIS	Histidine
ILE	Isoleucine
LEU	Leucine
LPS	Lipopolysaccharide
LYP	Lee-Yang-Parr
m/z	mass-to-charge ratio
MBC	Minimum Bactericidal Concentration
MD	Molecular Dynamics
MDA	Malondialdehyde
MET	Methionine
MHB	Mueller Hinton Broth
MIC	Minimum Inhibitory Concentration
MICs	Minimum Inhibitory Concentrations
NCTC	National Collection of Type Cultures
ND2	Amide nitrogen atom in the side chain of Asparagine
NIST	National Institute of Standards and Technology
O	Oxygen atom
O1	Oxygen atom of Thymoquinone
OG1	Hydroxyl oxygen atom in the side chain of Threonine
PDB	Protein Data Bank
PDB ID	Protein Data Bank Identifier
PHE	Phenylalanine
RMSD	Root Mean Square Deviation
THR	Threonine
TYR	Tyrosine
ΔG	Binding free energy
